# Targeted chemotherapy for subcutaneous and orthotopic non-small cell lung tumors with cyclic RGD-functionalized and disulfide-crosslinked polymersomal doxorubicin

**DOI:** 10.1038/s41392-018-0032-7

**Published:** 2018-12-14

**Authors:** Yan Zou, Jingjing Wei, Yifeng Xia, Fenghua Meng, Jiandong Yuan, Zhiyuan Zhong

**Affiliations:** 1 Biomedical Polymers Laboratory, College of Chemistry, Chemical Engineering and Materials Science, Soochow University, Suzhou, 215123 P. R. China; 2 International Joint Centre for Biomedical Innovation, School of Life Sciences, Henan University, Jin Ming Avenue, Kaifeng, Henan 475004 China; 3 BrightGene Bio-Medical Technology Co., Ltd, Suzhou, 215123 P. R. China

## Abstract

Lung cancer, with its high mortality and increasing morbidity, has become one of the most lethal malignancies worldwide. Here, we developed cyclic RGD peptide-directed and disulfide-crosslinked polymersomal doxorubicin (cRGD-PS-Dox) as a targeted chemotherapy for human non-small cell lung cancer (NSCLC). Notably, cRGD-PS-Dox exhibited a high Dox loading (15.2 wt.%), small hydrodynamic diameter (96 nm), superb stability, prominent targetability to α_v_β_3_ integrin overexpressing A549 human lung cancer cells, and rapid release of the drug into nuclei, leading to a significantly improved antitumor activity compared with the control groups, i.e., PS-Dox and Lipo-Dox (a liposome injection employed in clinical settings). The pharmacokinetic and biodistribution results for cRGD-PS-Dox revealed similar elimination half-lives but two-fold enhanced tumor accumulation compared with PS-Dox and Lipo-Dox. Intriguingly, cRGD-PS-Dox effectively suppressed the growth of A549 lung tumors in both subcutaneous and orthotopic models with minimal adverse effects at a Dox dose of 12 mg/kg, leading to significant survival benefits compared with PS-Dox and Lipo-Dox. This α_v_β_3_ integrin-targeting multifunctional polymersomal doxorubicin is highly promising for targeted chemotherapy of human NSCLC.

## Preclinical studies: Targeted lung cancer chemotherapy shows promise

When wrapped in an engineered vesicle and augmented with cancer-targeting peptides, chemotherapy drug doxorubicin shows increased efficacy in a preclinical study. Zhiyuan Zhong, from China’s Soochow University, and his team developed the therapeutic (cRGD-PS-Dox) that targets cancer cells that overexpress a specific protein (α_v_β_3_ integrin), such as those of non-small cell lung cancer. *In vitro* assays showed that cRGD-PS-Dox specifically targeted and inhibited cancer cells, and inhibited the growth and metastasis of human tumor grafts in mice. *In vivo* imaging confirmed a desirable drug stability profile and accumulation within tumors. These results showed clear advantages over non-targeted doxorubicin treatment controls. Mice treated with cRGD-PS-Dox also survived significantly longer than control-treated mice. The preferential attributes of the therapy make it a promising agent for further study into tumors that overexpress α_v_β_3_ integrin.

## Introduction

Lung cancers have high mortality and increasing morbidity and have become one of the most lethal malignancies worldwide.^1–3^ Despite the severe side-effects, chemotherapy with potent drugs such as erlotinib, gemcitabine, cisplatin, irinotecan, doxorubicin hydrochloride (Dox·HCl), and pemetrexed continues to serve as the primary treatment and supportive care for lung cancer patients.^4–6^ During the past decade, diverse anticancer nanomedicines have been investigated to increase anticancer efficacy while decreasing the adverse effects of chemotherapeutic drugs.^7–18^ In a phase II clinical trial, Genexol-PM, a micellar paclitaxel formulation, in combination with gemcitabine, demonstrated favorable antitumor activity in non-small cell lung cancer (NSCLC) patients.^19^ To augment the tumor targetability, nano-drugs have been decorated with lung cancer cell-specific ligands, such as anisamide and peptides (e.g., cNGQGEQ and CSNIDARAC).^20–23^ Nonetheless, these “targeted” systems show only a moderate improvement in therapeutic efficacy, partially due to low in vivo stability and/or slow release of drugs intracellularly. Interestingly, disulfide-crosslinked nanomedicines have demonstrated excellent in vivo stability and fast intracellular drug release.^24–28^ We have described that disulfide-crosslinked chimeric polymersomes containing poly(ethylene glycol) on the outer surface and polyethylenimine mainly in the lumen efficiently mediated targeted delivery of methotrexate sodium (MTX·2Na) to lung tumor xenografts in nude mice.^29^

**Fig. 1:**
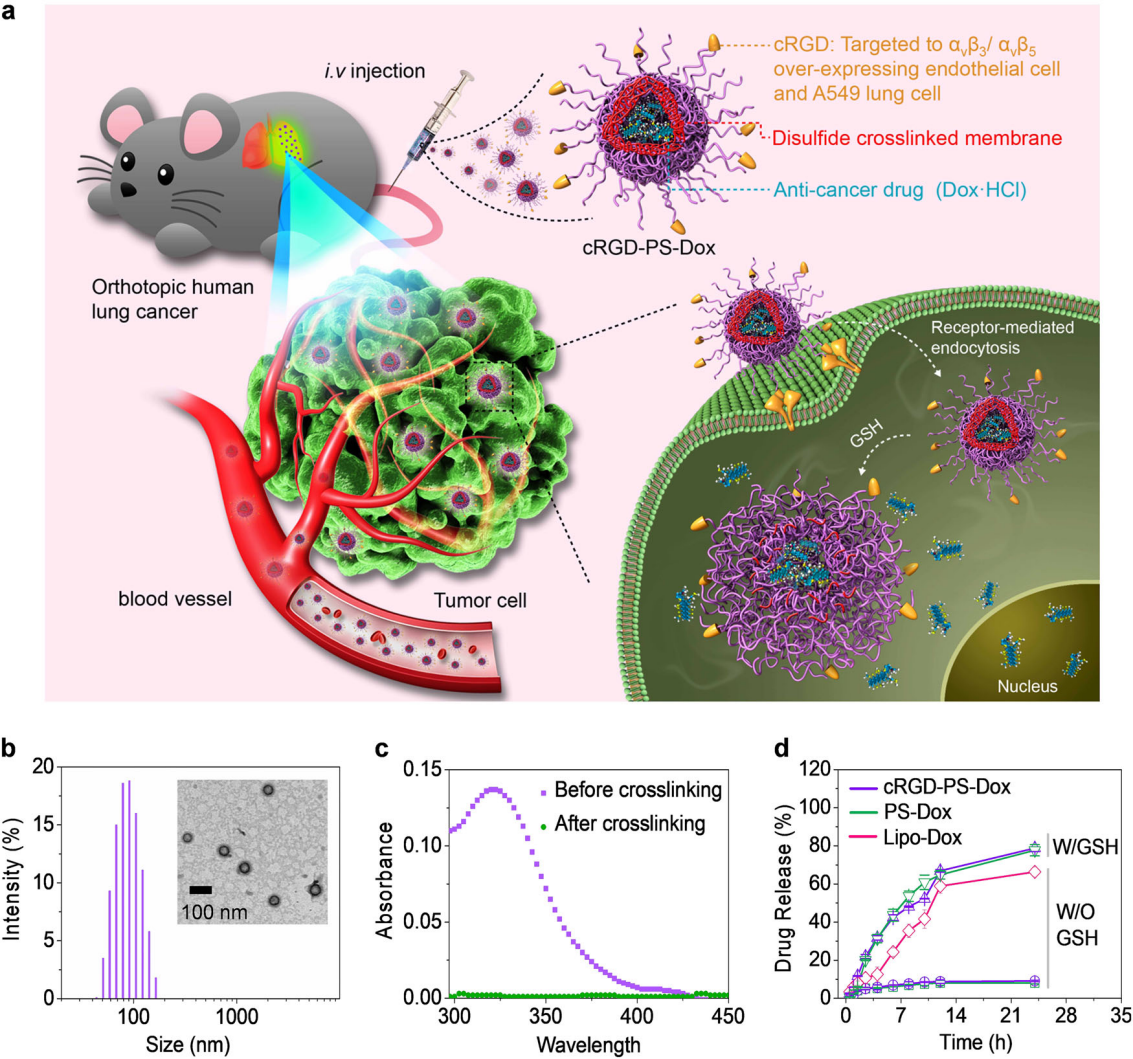
**a** Illustration of cRGD-directed polymersomal doxorubicin (cRGD-PS-Dox) for active targeting chemotherapy of lung tumor xenografts in mice; **b** DLS measurement and TEM photograph of cRGD-PS; **c** UV absorbance of cRGD-PS before and after crosslinking; **d** In vitro Dox release in PB with or without GSH (10 mM) at 37 °C

Here, we report on cRGD peptide-directed and disulfide-crosslinked polymersomal doxorubicin (cRGD-PS-Dox) for active targeting chemotherapy of human lung cancer xenografts in mice (Fig. 1a). PS-Dox has a vesicular structure, small size, and high loading of Dox·HCl,^21^ similar to pegylated liposomal doxorubicin (Doxil or Caelyx) used in the clinic.^30–35^ cRGD was selected as a targeting ligand because both angiogenic endothelial cells and A549 human lung cancer cells overexpress α_v_β_3_ integrins.^36–40^ Our results show that cRGD-PS-Dox effectively suppresses the growth of A549 lung tumors in both subcutaneous and orthotopic models in mice, resulting in clear survival benefits over PS-Dox and Lipo-Dox.

## Results

### Construction of cRGD-PS-Dox

Small cRGD-PS (~82 nm) with a low polydispersity index (PDI = 0.10) was obtained by co-self-assembly of poly(ethylene glycol)-*b*-poly(trimethylene carbonate-*co*-dithiolane trimethylene carbonate) (PEG-P(TMC-DTC), *M*_n_ = 5.0–24.2 kg/mol) and cRGD-functionalized PEG-P(TMC-DTC) (cRGD-PEG-P(TMC-DTC), *M*_n_ = 6.5–24.5 kg/mol) block copolymers (4/1, mol/mol) in phosphate buffer (PB, pH 7.4, 10 mM) (Fig. 1b). Transmission electron microscope (TEM) micrograph corroborated a vesicular morphology and small size for cRGD-PS (Fig. 1b, inset). UV spectra showed a significant decrease in the characteristic absorbance of dithiolanes at 330 nm in cRGD-PS (Fig. 1c), signifying that dithiolanes underwent spontaneous ring-opening polymerization forming linear polydisulfides that crosslinked the vesicular membrane. Accordingly, cRGD-PS-Dox and PS-Dox did not have a critical vesicle concentration (CVC).

As previously reported, Dox·HCl could be laden into PS by a pH-gradient method with high efficiency,^41^ and a drug loading content (DLC) of 15.2 wt.% was achieved for cRGD-PS-Dox, which exhibited a hydrodynamic diameter of 96 nm (PDI = 0.13, Table 1). Notably, less than 15% of drug was released from cRGD-PS-Dox in 24 h at pH 7.4 and 37 °C under 2 µM GSH (Figure S1), indicating a high stability of cRGD-PS-Dox in circulation. The Dox release from cRGD-PS-Dox increased to approximately 46%, 62%, and 80% upon increasing GSH concentrations to 2, 5 and 10 mM, respectively (Fig. 1d & Figure S1). In comparison, 67% Dox was released from Lipo-Dox in 24 h under physiological conditions. These results suggest that cRGD-PS-Dox might be robust in the circulation while quickly releasing drug in the cytoplasm.

**Table 1:** Characteristics of cRGD-PS-Dox and PS-Dox

Polymersomes	DLC^a^ (wt.%)				
	Theory	Determined	DLE^a^ (%)	Size^b^ (nm)	PDI^b^
PS-Dox	9.1	6.7	72.1	85	0.08
	16.7	12.0	68.4	88	0.11
	23.1	15.5	61.3	94	0.13
cRGD-PS-Dox	9.1	6.8	73.5	82	0.06
	16.7	11.8	67.2	86	0.12
	23.1	15.2	59.6	96	0.13

^a^Measured by fluorometry

^b^Measured by DLS at 25 °C in PB (polymersome concentration: 1.0 mg/mL)

### In vitro assessment of cRGD-PS-Dox

**Fig. 2:**
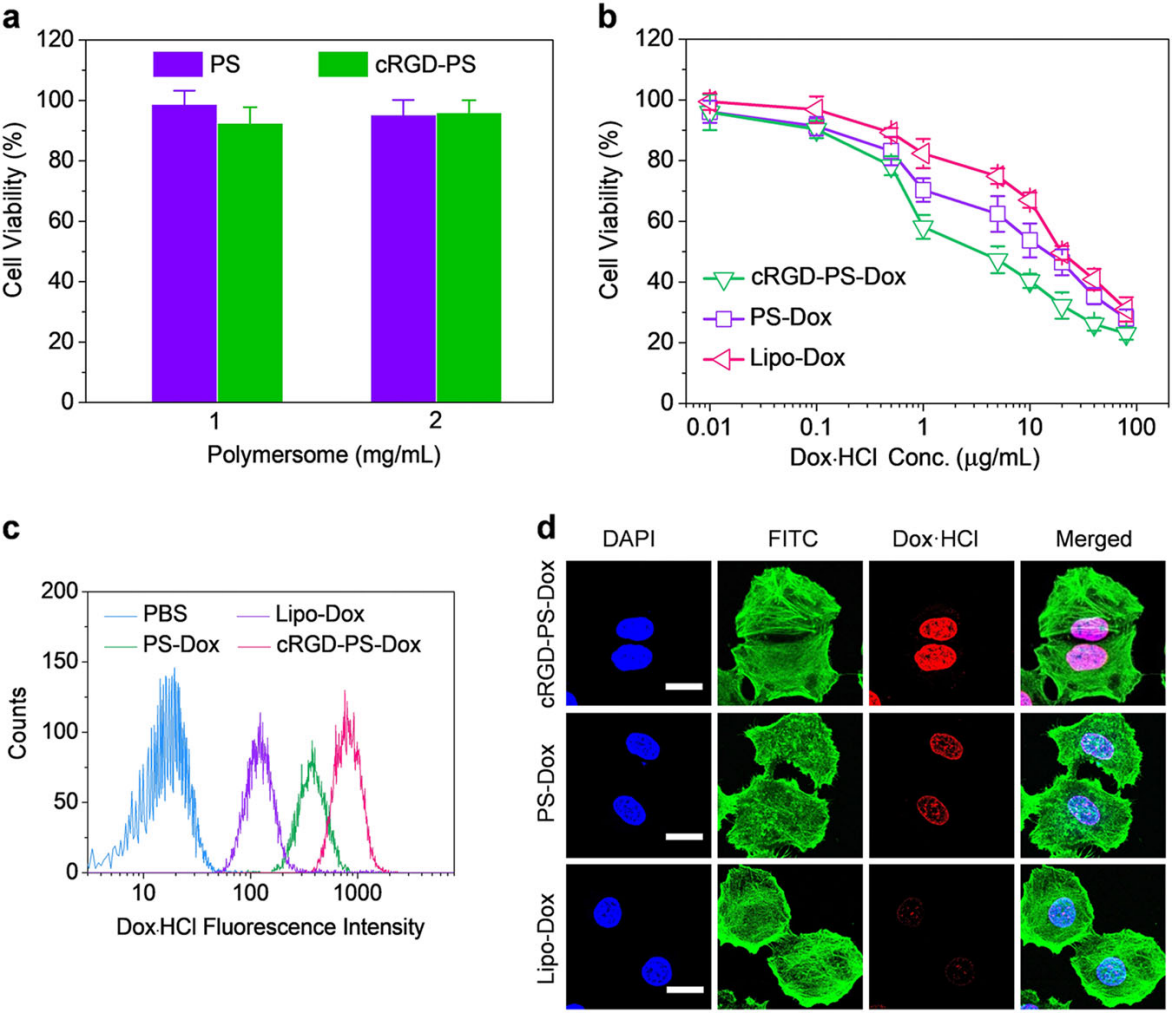
**a** Cytotoxicity of blank cRGD-PS to A549 cells following 48 h incubation. **b** Viability of A549 cells after a 4 h incubation with cRGD-PS-Dox plus 44 h culture in fresh media. **c** Flow cytometry of A549 cells after a 4 h incubation with different formulations (10.0 μg/mL Dox·HCl). **d** CLSM of A549 cells treated with different formulations for 4 h (10.0 μg/mL Dox·HCl) and further cultured in fresh media for 4 h. The cell nuclei and cytoskeleton were stained by DAPI and phalloidin-FITC, respectively. Scale bars: 25 μm

MTT assays demonstrated that blank polymersomes, either targeted or non-targeted, were non-toxic to α_v_β_3_ integrin-overexpressing human lung cancer A549 cells at tested concentrations (1 and 2 mg/mL, Fig. 2a), signifying their low cytotoxicity. In contrast, cRGD-PS-Dox exhibited potent inhibitory effects on A549 cells with an IC_50_ of 3.2 μg/mL, which was 4.1- and 6.4-fold lower than PS-Dox and Lipo-Dox, respectively (Fig. 2b). Notably, a comparable cytotoxicity profile was discerned for cRGD-PS-Dox and PS-Dox in α_v_β_3_ integrin low-expressing human MCF-7 breast cancer cells (Figure S2a). Flow cytometric analyses confirmed that the intracellular Dox·HCl intensity in cRGD-PS-Dox treated A549 cells was stronger compared with those of PS-Dox or Lipo-Dox (Fig. 2c). In MCF-7 cells, however, cRGD-PS-Dox exhibited similar cellular uptake to PS-Dox (Figure S2b), verifying that cRGD-PS-Dox has a high specificity to α_v_β_3_ integrin-overexpressing cancer cells. CLSM observation displayed intensive Dox·HCl fluorescence in the nuclei of A549 cells treated with cRGD-PS-Dox (Fig. 2d). In contrast, PS-Dox showed weak fluorescence in the nuclei and Lipo-Dox showed negligible fluorescence. The enhanced nucleic Dox release observed for cRGD-PS-Dox is likely due to its efficient uptake by A549 cells via a receptor-mediated mechanism and triggered drug release in the cytoplasm.

### In vivo pharmacokinetics and biodistribution of cRGD-PS-Dox

**Fig. 3:**
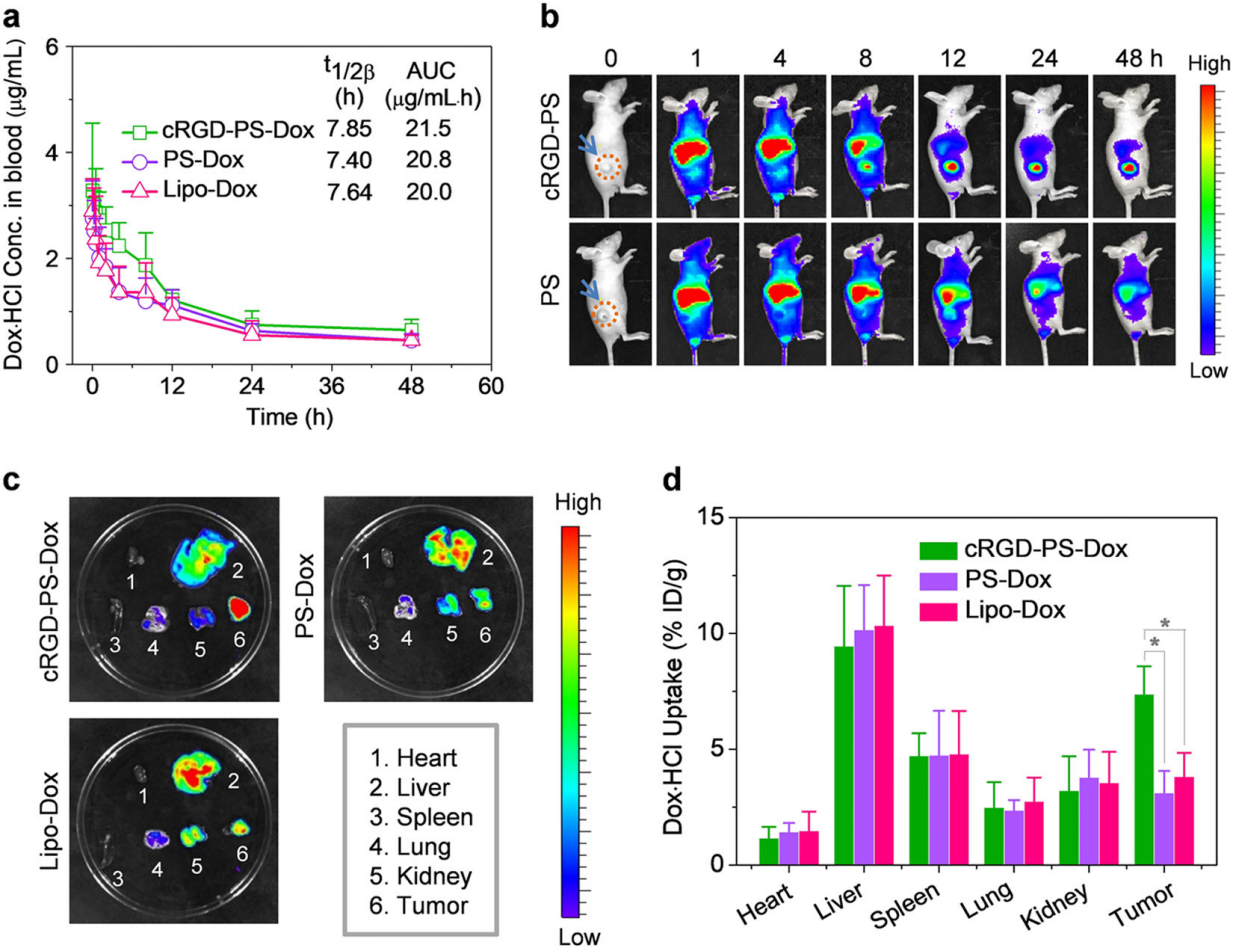
**a** In vivo pharmacokinetics of cRGD-PS-Dox, PS-Dox and Lipo-Dox in tumor-free mice. **b** In vivo fluorescence images of mice bearing subcutaneous A549 tumors after *iv* injection of Cy7-labeled cRGD-PS or PS. **c** Ex vivo fluorescence images of tumors and major organs isolated at 12 h post-injection. **d** Quantitative drug biodistribution in tumors and major organs. *p *<* 0.05 based on one-way ANOVA and Tukey multiple comparisons tests. For **a** and **d**, the Dox·HCl concentration was measured by fluorescence spectroscopy and are presented as the mean ± SD (*n* = 3)

In vivo stability is vital for tumor accumulation and the anti-tumor efficacy of nanotherapeutics.^42,43^ The Dox·HCl concentration in plasma was monitored over time after a single injection of cRGD-PS-Dox or PS-Dox into healthy mice. The results showed that they had an elimination half-lives (t_1/2,β_) of approximately 7.5 h and a high area under curve (AUC) comparable to that of Lipo-Dox (Fig. 3a). To visualize their tumor accumulation, Cy7-labeled cRGD-PS or PS was injected into nude mice inoculated with A549 tumor xenografts subcutaneously and observed over time using an IVIS system. Figure 3b showed that tumor accumulation of cRGD-PS became obvious at 1 h post-injection, reached a maximum at 12 h and remained high at 48 h. Notably, the liver uptake reduced to a low level after 24 h. In comparison, Cy7-labeled PS accumulated significantly less at tumor sites despite the similar pharmacokinetics of cRGD-PS and PS, indicating the decisive role of active targeting in tumor accumulation, cell uptake and retention.

Taking advantage of the fluorescent nature of free Dox, we then investigated the biodistribution of cRGD-PS-Dox by ex vivo fluorescence imaging. The tumor and major organs were excised at 12 h post-injection and imaged using a fluorescence imaging system. Figure 3c shows that for cRGD-PS-Dox treated mice, tumors had more intense Dox·HCl fluorescence than healthy organs, supporting selective uptake by A549 tumors, rapid cellular internalization and rapid Dox release. In sharp contrast, PS-Dox and Lipo-Dox groups showed comparably weaker Dox·HCl fluorescence in tumors. The quantitative measurements of Dox·HCl demonstrated that tumor accumulation of cRGD-PS-Dox could reach approximately 7.37% of the injected dose per gram of tissue (% ID/g), which was approximately 1.9-fold better than the Lipo-Dox group and 2.4-fold better than the PS-Dox group (Fig. 3d). The summary of tumor-to-normal tissue (T/N) ratios revealed that cRGD-PS-Dox treatment significantly reduced the Dox·HCl amount in major organs compared to Lipo-Dox and PS-Dox (Table S1). The above results suggest that cRGD functionalization of PS-Dox remarkably enhances tumor accumulation and retention.^44,45^ The tolerability studies revealed that cRGD-PS-Dox did not cause significant body weight loss at a Dox·HCl dose of 100 or 150 mg/kg while Lipo-Dox induced high toxicity at 20 mg/kg (Figure S3), indicating that cRGD-PS-Dox has at least a 7.5-fold higher maximum-tolerated dose (MTD) than Lipo-Dox.

### In vivo therapeutic administration of nude mice bearing subcutaneous A549 tumor xenografts

**Fig. 4:**
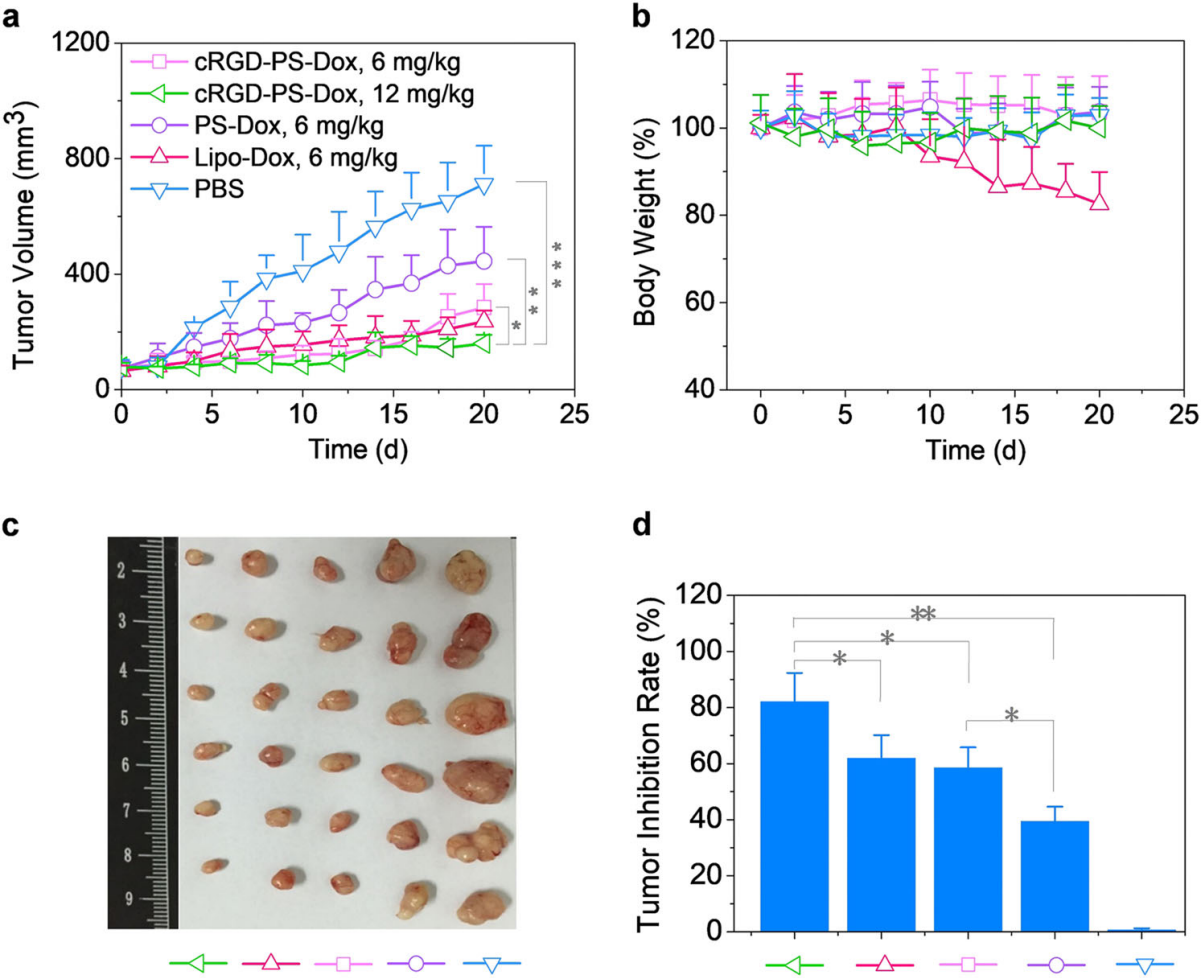
Antitumor activity of cRGD-PS-Dox and PS-Dox in nude mice bearing subcutaneous A549 tumors. The drug was given on day 0, 4, 8, and 12 (Dox dose: 6 or 12 mg/kg, in 200 µL PBS). Lipo-Dox and PBS were used as controls. **a** Tumor volumes over 20 days. **b** Body weight changes over 20 d. **c** Photographs of tumor blocks collected on day 20. **d** Tumor inhibition rate on day 20. *p < 0.05, **p < 0.01 and ***p < 0.001 based on one-way ANOVA and Tukey multiple comparisons tests

In vivo antitumor activity of cRGD-PS-Dox was assessed in mice bearing subcutaneous A549 xenografts at a Dox·HCl equivalent dose of 6 or 12 mg/kg administered on day 0, 4, 8, and 12. The results demonstrated a dose-dependent tumor growth inhibition by cRGD-PS-Dox. Tumor progression was greatly suppressed at 12 mg/kg (Fig. 4a). At 6 mg/kg, cRGD-PS-Dox exhibited similar efficacy to Lipo-Dox while significantly better tumor suppression than PS-Dox, suggesting that cRGD-PS-Dox can actively target A549 tumors in vivo. Figure 4b reveals that Lipo-Dox caused severe systemic toxicity with substantial loss in body weight (up to 18%) during treatment. In contrast, for cRGD-PS-Dox and PS-Dox treated mice, their body weights had little alteration, indicating that polymersomal Dox has negligible adverse effects. The pictures of tumors collected on day 20 confirmed that mice treated with cRGD-PS-Dox at 12 mg/kg bore the smallest tumors (Fig. 4c). Figure 4d demonstrates that cRGD-PS-Dox had a remarkable tumor inhibition rate (TIR) of 82.4% at 12 mg/kg. Moreover, at 6 mg/kg cRGD-PS-Dox exhibited a TIR similar to Lipo-Dox though significantly higher than PS-Dox. These results clearly show that cRGD-PS-Dox has a markedly improved safety profile, tumor targetability and therapeutic efficacy over Lipo-Dox in A549 tumor-bearing nude mice.

### In vivo treatment of nude mice bearing orthotopic A549-Luc tumor xenografts

**Fig. 5:**
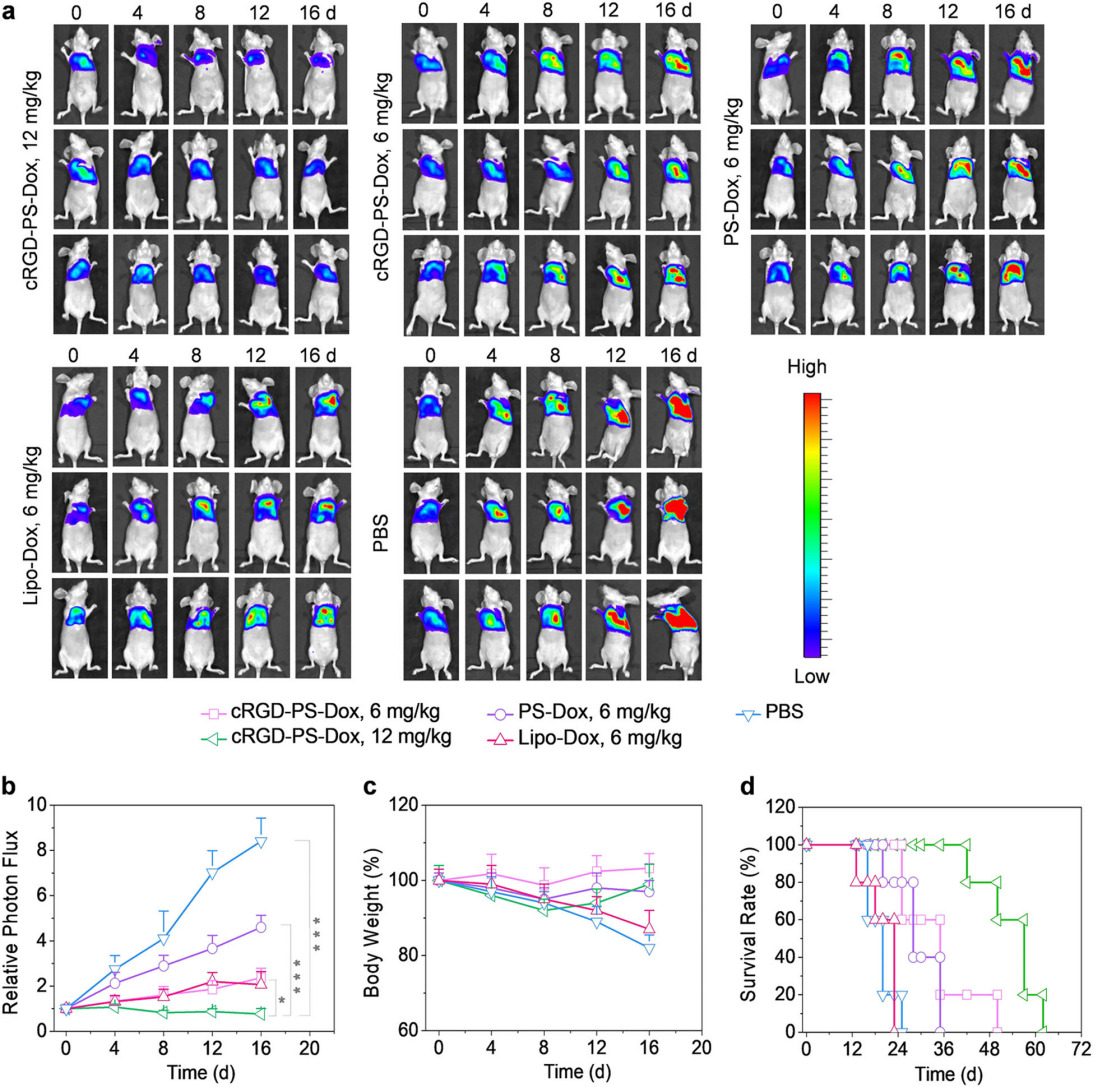
In vivo antitumor activity of cRGD-PS-Dox, PS-Dox, and Lipo-Dox in nude mice bearing orthotopic A549 tumor xenografts at a Dox dose of 6 or 12 mg/kg in 200 µL PBS administered on day 0, 4, 8, and 12. **a** Bioluminescence images of mice over time. **b** Bioluminescence intensity of mice. One-way ANOVA and Tukey multiple comparisons tests, *p *<* 0.05 and ***p < 0.001. **c** Body weight of mice over 16 days. **d** Survival curves of mice

**Fig. 6:**
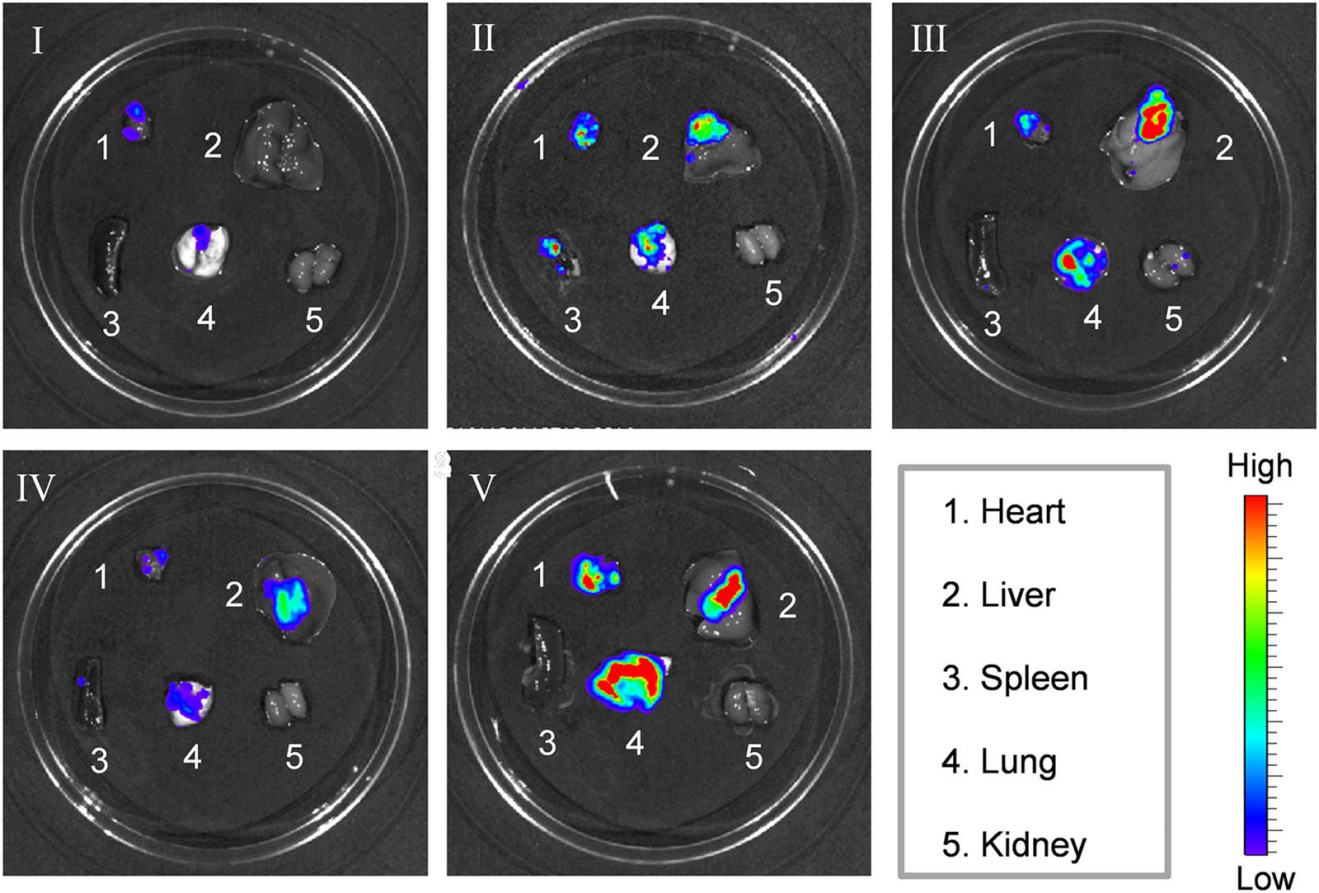
Ex vivo luminescence images of major organs from mice bearing orthotopic A549-Luc tumors on day 16. I. cRGD-PS-Dox@12 mg/kg, II. cRGD-PS-Dox@6 mg/kg, III. PS-Dox@6 mg/kg, IV. Lipo-Dox@6 mg/kg, V. PBS

The exceptional therapeutic benefits of cRGD-PS-Dox in the treatment of subcutaneous lung tumor models encouraged us to further assess its targetability and antitumor effects in orthotopic human A549 lung tumor xenografts in mice, which was established by implanting bioluminescent A549-Luc cancer cells into the lungs of nude mice (*n* = 6). The growth of tumors was monitored using the IVIS imaging system. All of the mice had similar luminescence intensity initially, and cRGD-PS-Dox (12 mg/kg) induced the best tumor inhibition over a treatment period of 16 days. At 6 mg/kg, cRGD-PS-Dox resulted in better tumor suppression than PS-Dox, as observed in the subcutaneous model (Fig. 5a). The quantification of lung bioluminescence intensity showed that cRGD-PS-Dox (12 mg/kg) completely suppressed tumor growth. cRGD-PS-Dox led to similar tumor inhibition as Lipo-Dox while cRGD-PS-Dox performed significantly better than PS-Dox (Fig. 5b). Notably, the body weights only slightly changed for cRGD-PS-Dox or PS-Dox treated mice while Lipo-Dox and PBS groups displayed significant body weight loss over 16 days **(**Fig. 5c**)**, confirming that cRGD-PS-Dox has few side effects and can effectively retard tumor invasion into the lung. Consistently, cRGD-PS-Dox (12 mg/kg) treated mice survived the longest with a median survival time of 57 days (Fig. 5d). Meanwhile, at 6 mg/kg, cRGD-PS-Dox also exhibited a clear survival benefit over Lipo-Dox and PS-Dox. On day 16, the major organs were collected from one mouse in each group for ex vivo imaging, in which the lowest bioluminescence was observed in the lung of the mouse treated with 12 mg/kg cRGD-PS-Dox (Fig. 6a). Of note, no metastasis to the liver was observed with 12 mg/kg cRGD-PS-Dox, which is in great contrast to the mice treated with the other formulations. cRGD-PS-Dox at 12 mg/kg can effectively target and suppress the growth and metastasis of orthotopic A549 lung tumors with few adverse effects, outperforming PS-Dox and Lipo-Dox.

## Discussion

Lung cancer is a rapidly growing human malignancy. cRGD induces significantly enhanced imaging of lung tumors via binding to α_v_β_3_ integrins.^46–48^ In this study, we investigated the targetability and anti-tumor activity of cRGD-PS-Dox in mice with subcutaneous or orthotropic A549 tumors. The pharmacokinetics studies demonstrated that cRGD-PS-Dox can circulate in the blood, comparable to Lipo-Dox. The biodistribution of cRGD-PS-Dox reveals a much higher drug accumulation in tumors (7.37% ID/g) than that of PS-Dox or Lipo-Dox, validating that cRGD functionalization enables active targeting to A549 tumors in vivo.^40,49^ Interestingly, cRGD-PS-Dox exhibits an extraordinarily high MTD (over 150 mg/kg) that is over 7.5-fold higher than that of Lipo-Dox. Therefore, cRGD-PS-Dox has a much broader therapeutic window compared with Lipo-Dox. A narrow therapeutic window is a key problem for chemotherapeutics and cancer nanomedicines, including Lipo-Dox.^50,51^ The in vivo studies in subcutaneous and orthotropic A549 lung tumor-bearing mice demonstrated that cRGD-PS-Dox can efficiently suppress tumor progression, significantly improve survival rates, and largely reduce side effects compared to Lipo-Dox and PS-Dox. Notably, when treated with 12 mg/kg cRGD-PS-Dox, the mice inoculated with orthotopic A549 lung tumors exhibited a median survival time of 57 days, which was significantly longer than that of Lipo-Dox. The high potency of cRGD-PS-Dox can be attributed to its long circulation time, efficient and specific uptake by α_v_β_3_ integrin-overexpressing A549 cells, and triggered release of Dox in the cytoplasm, which contains 2–10 mM GSH, leading to a greatly enhanced nucleic delivery of Dox. Dox is known to induce antitumor effects through intercalation into the DNA of cancer cells.^52,53^

In conclusion, we established that polymersomal doxorubicin cRGD-PS-Dox achieves an ultrahigh treatment efficacy in mice bearing subcutaneous or orthotropic human lung cancers with few adverse effects. Of note, cRGD-PS-Dox has several unique and desirable merits, such as high drug loading, a small size, superb stability, exceptional tolerability, high tumor accumulation and targetability, and modulated drug release. This polymersomal doxorubicin holds promise as a therapeutic agent for treating various α_v_β_3_/α_v_β_5_ integrin-overexpressing tumors.

## Materials and methods

### In vivo pharmacokinetics of cRGD-PS-Dox

The mice were handled under protocols approved by Soochow University Laboratory Animal Center and the Animal Care and Use Committee of Soochow University. Tumor-free nude mice (18–20 g, *n* = 3) were *i.v*. injected with cRGD-PS-Dox (Dox dose: 10 mg/kg) in 200 μL PBS. Lipo-Dox, PS-Dox and PBS were used as controls. At set time points, approximately 50 μL of blood was withdrawn from the eye sockets of mice, and Triton X-100 (1 wt%, 0.1 mL) was added immediately with brief sonication. To extract Dox·HCl, 0.5 mL dimethyl formamide (DMF) solution containing 20 mM dithiothreitol (DTT) was added, incubated for 6 h and stored at −20 °C overnight. After centrifugation (14.8k rpm, 15 min), the supernatants were measured using fluorometry for Dox·HCl concentration. The blood circulation curves were drawn, and the half-lives were calculated as reported previously.^21,41^

### Imaging of cRGD-PS-Dox in subcutaneous tumor xenografts

Briefly, a subcutaneous A549 tumor model was established by injecting A549 cells (1 × 10^7^ cells in 50 μL PBS) into the right hind flank of nude mice (female, 18–20 g). The tumor size reached approximately 150 mm^3^ after 15 days. For monitoring polymersomes in mice using fluorescence imaging, Cy7-labeled cRGD-PS and PS in 200 μL PB, prepared by incorporating 5 mol% Cy7-labeled PEG-P(TMC-DTC) copolymer, were *i.v*. injected into randomly grouped tumor-bearing mice via tail vein. After 0, 1, 4, 8, 12, 24 or 48 h, the mice were anesthetized and immediately imaged with an IVIS Lumina II with constant isoflurane (3%) supply.

### Biodistribution of cRGD-PS-Dox

A subcutaneous A549 tumor xenograft model was established as described above. cRGD-PS-Dox, PS-Dox, or Lipo-Dox (Dox·HCl dose: 10 mg/kg) or PBS was *i.v*. injected into randomly grouped mice bearing A549 subcutaneous tumor xenografts (*n* = 3). After 12 h, tumors and major organs were excised, flushed, towel-dried and weighed before being subjected to fluorescence imaging.

The tumors and organs were subsequently homogenized (IKA T25, 18k rpm, 10 min) in 600 µL Triton X-100 solution (1%). An extraction solution (900 µL DMF with 20 mM DTT and 50 mM HCl) was added to each tissue lysate, incubated for 6 h and stored at −20 °C overnight. After centrifugation, the supernatants were measured using fluorometry for Dox·HCl concentration, which was expressed as injected dose per gram of tissue (%ID/g).

### In vivo antitumor performance of cRGD-PS-Dox in subcutaneous lung tumor model

A mouse subcutaneous A549 tumor xenograft model was established as described above. Treatment started when the tumor volume reached 100 mm^3^ after 10 days, defined as day 0. The mice were randomly assigned into five groups (*n* = 6): cRGD-PS-Dox (6 or 12 mg/kg), PS-Dox (6 mg/kg), Lipo-Dox (6 mg/kg) and PBS. The formulations and control were intravenously injected every 4 days (total 4 injections). The body weight and tumor size of the mice were measured every 2 days and normalized to their initial values on day 0. On day 20, the mice were sacrificed and tumors were excised and weighed. The tumor inhibition rates were calculated compared to the tumor weight of the PBS treated group.

### In vivo antitumor efficacy of cRGD-PS-Dox in an orthotopic lung tumor model

A549-Luc cancer cells (1 × 10^7^ in 100 μL PBS) were injected into the lungs of female nude mice (18–20 g) through syringes. Tumor development was estimated using an IVIS Lumina system by visualizing cancer cells transfected by luciferase at 10–15 min after the injection of luciferin intraperitoneally (150 mg luciferin/kg of body weight). Normally after 10 days, the tumor luminescent region of interest (ROI) intensity of the mice reached approximately 1 × 10^4^–2 × 10^4^. The grouping and dosing of the mice were the same as in the subcutaneous lung tumor model. The day treatment started was defined as day 0. The mice were weighed every four days and normalized to their initial weights on day 0. Every four days, the tumor bioluminescence was tracked using the IVIS Lumina system, and the relative photon flux was normalized to the initial intensity, I/I_0_ (I_0_ is the bioluminescent intensity on day 0). All images were set to the same conditions and color scale. On day 16, one mouse from each group was sacrificed after injection of luciferin. Major organs were excised, washed and imaged by an IVIS II instrument. The remaining mice were observed for survival curves (*n* = 5).

### Statistical analysis

Differences between groups were assessed using the one-way ANOVA and Tukey multiple comparisons tests. Kaplan-Meier survival curves were analyzed by one-way ANOVA with a log-rank test for comparisons. Data were analyzed using GraphPad Prism 7 and are expressed as the mean ± SD. *p *<* 0.05 was considered significant, and **p *<* 0.01, ***p < 0.001 were considered highly significant.

## Electronic supplementary material

Supporting information
