# Supplementary material for: Targeted chemotherapy for subcutaneous and orthotopic non-small cell lung tumors with cyclic RGD-functionalized and disulfide-crosslinked polymersomal doxorubicin
Source: Signal Transduct Target Ther. 2018 Dec 14;3:32. doi: 10.1038/s41392-018-0032-7 (PMC6292884; doi:10.1038/s41392-018-0032-7)
Supplement: Supplementary file 1 — Supporting information [file 41392_2018_32_MOESM1_ESM.doc]

Supporting Information

**Targeted chemotherapy for subcutaneous and orthotopic non-small cell lung tumors with cyclic RGD-functionalized and disulfide-crosslinked polymersomal doxorubicin**

Yan Zou^1,2^, Jingjing Wei^1^, Yifeng Xia^1^, Fenghua Meng^1^,*, Jiandong Yuan^3^, and Zhiyuan Zhong^1^,*

^1^Biomedical Polymers Laboratory, and Jiangsu Key Laboratory of Advanced Functional Polymer Design and Application, College of Chemistry, Chemical Engineering and Materials Science, Soochow University, Suzhou, 215123, P. R. China.

^2^International Joint Centre for Biomedical Innovation, School of Life Sciences, Henan University, Jin Ming Avenue, Kaifeng, Henan, 475004, China

^3^BrightGene Bio-Medical Technology Co., Ltd., Suzhou, 215123, P. R. China

*Corresponding authors. Tel/Fax: +86-512-65880098, Email: [fhmeng@suda.edu.cn](mailto:fhmeng@suda.edu.cn) (F. Meng); [zyzhong@suda.edu.cn](mailto:zyzhong@suda.edu.cn) (Z. Zhong)

**Materials**

Poly(ethylene glycol) monomethyl ether (MeO-PEG-OH, *M*_n_ = 5.0 kg/mol, PDI = 1.03, Fluka) was dried by azeotropic distillation from anhydrous toluene before use. Dichloromethane (DCM) was dried by refluxing over CaH_2_. Trimethylene carbonate (TMC) was recrystallized over dry toluene. Diphenyl hydrogen phosphate (DPP, 98%, TCI) was dried before use. N-hydroxysuccinimide activated poly(ethylene glycol) (NHS-PEG-OH, *M*_n_ = 6.5 kg/mol, 96.8%, PDI = 1.04, Beijing JenKem Technology Co., Ltd.), cyclic peptide C(RGDfK) (cRGD, 98%, China Peptides Co., Ltd), glutathione (GSH, 99%, Roche), dithiothreitol (DTT, 99%, Merck), Cy7-NHS (Beijing Fanbo biochemicals), doxorubicin hydrochloride (Dox·HCl, > 99%, Beijing Zhongshuo Pharmaceutical Technology Development), 3-(4,5-dimethylthiazol-2-yl)-2,5-diphenyltetrazolium bromide (MTT, Sigma), Triton X-100 (Sigma), and isoflurane (Abbott laboratories, Chicago, IL) were used as received. Bioluminescent human large cell lung cancer luciferase cells (A549-Luc) were obtained from Shanghai Lechen Biotech. Co. Ltd.. PEG-P(TMC-DTC) (*M*_n_ = 5.0-(22.4-2.0) kg/mol) and cRGD-PEG-P(TMC-DTC) (6.5-(22.6-1.9) kg/mol) copolymers were synthesized as reported previously.^1^

**Characterization**

The size and size distribution of polymersomes were determined at 25 °C using dynamic light scattering (DLS, Zetasizer Nano-ZS, Malvern Instruments) equipped with a 633 nm He–Ne laser using back-scattering detection. The measurements were performed in triplicate. Transmission electron microscopy (TEM) was performed using a Tecnai G220 TEM operated at an accelerating voltage of 120 kV. Dropping 10 μL of 0.2 mg/mL polymersome suspension on the copper grid followed by staining with phosphotungstic acid (1 wt.%) to prepare the TEM samples. The CLSM images of cells were acquired by a confocal laser scanning microscope (TCS SP5). The in vivo fluorescence images were acquired using a near-infrared fluorescence imaging system (Caliper IVIS Lumina II).

**Preparation of cRGD-decorated disulfide-crosslinked polymersomes (cRGD-PS)**

cRGD-decorated crosslinked polymersomes (cRGD-PS) were co-self-assembled from PEG-P(TMC-DTC)/cRGD-PEG-P(TMC-DTC) via solvent exchange method. Briefly, 900 μL phosphate buffer (PB, 10 mM, pH 7.4) was dropwise added to 100 μL mixed DMF solution of PEG-P(TMC-DTC) and cRGD-PEG-P(TMC-DTC) (10.0 mg/mL, w/w, 4/1), followed by extensive dialysis against PB using a dialysis bag (Spectra/Pore, MWCO 7000) for 12 h. The dialysis medium was refreshed each hour. The size and size distribution were measured by DLS.

**Loading and reduction-triggered release of Dox·HCl**

Dox·HCl was loaded into polymersomes by pH-gradient method as our previous reports.^2, 3^ Briefly, 0.5 mL DMF solution of PEG-P(TMC-DTC) and cRGD-PEG-P(TMC-DTC) mixture (10.0 mg/mL, w/w, 4/1) was slowly added to 4.5 mL of citric acid buffer (10 mM, pH 4.0). After standing at room temperature for 5 h, the solution pH value was quickly adjusted to 7.8 via supersaturated disodium hydrogen phosphate solution. Thus formed polymersomes were divided into three aliquots, to which were added 20, 40 and 60 μL of Dox·HCl solution in distilled water (5 mg/mL), corresponding to a theoretical drug loading content (DLC) of 9.1, 16.7 and 23.1 wt.%, respectively. Following 5 h stirring at 37 °C, the polymersomes were extensively dialyzed against PB (10 mM, pH 7.4) to remove free Dox·HCl. The dialysis medium was changed per hour for at least 8 times and the whole procedure was performed in the dark. To quantify the drug loading content (DLC) and drug loading efficiency (DLE), cRGD-PS-Dox suspension was diluted with DMF (30-fold), sonicated and analyzed with fluorometry with excitation at 480 nm and emission at 560 nm. Calibration curve was obtained with a series of Dox·HCl solution with known concentrations in DMF, DLC and DLE were calculated according to the following formula:

DLC (*wt.*%) = (weight of loaded drug/total weight of loaded drug and polymer) × 100

DLE (%) = (weight of loaded drug/weight of drug in feed) ×100.

The *in vitro* Dox·HCl release of cRGD-PS-Dox was studied using a dialysis tube (Spectra/Pore, MWCO 12000-14000) at a polymersome concentration of 50 mg/L, in PB (10 mM, pH 7.4) or PB (10 mM, pH 7.4) containing 0.002, 2, 5, or 10 mM GSH, in shaking bed (37 °C, 200 rmp). Lipo-DOX was used as control. Typically, 600 µL of cRGD-PS-Dox and PS-Dox were dialyzed against 25 mL of the corresponding medium to obtain sink conditions. At desired time intervals, 5.0 mL of release medium was taken out and replenished with an equal volume of fresh medium. The amount of released Dox·HCl was quantified using fluorometry (Agilent technologies, cary Eclips fluorescence spectrophotometer) based on a calibration curve. The release experiment was operated in triplicate, and the results presented were the average data with standard deviations.

***In vitro* cytotoxicity assays**

The cytotoxicity of cRGD-PS and antitumor activity of cRGD-PS-Dox were assessed using MTT assays on α_v_β_3_/α_v_β_5_ integrin-overexpressing A549 human lung cancer cells. α_v_β_3_/α_v_β_5_ Integrin low-expressing human MCF-7 breast cancer cells were used as a negative control. The cells were seeded in 96-well plate at a density of 5 × 10^3^ cells/well in RMPI 1640 medium containing 10% fetal bovine serum (FBS), antibiotics penicillin (100 IU/mL) and streptomycin (100 μg/mL) for 24 h. 10 μL of cRGD-PS-Dox, PS-Dox or Lipo-Dox (Dox·HCl concentrations ranging from 0.001 to 80 μg/mL) in PBS was added. The cells were incubated in an atmosphere containing 5% CO_2_ at 37 °C for 4 h. The media were removed and replaced by fresh medium, and the cells were cultured for another 44 h. Subsequently, 10 μL of 3-(4,5-dimethylthiazol-2-yl)-2,5-diphenyl tetrazoliumbromide (MTT) solution (5 mg/mL) was added to each well. The cells were incubated for 4 h, the culture medium was discarded, and 150 μL of DMSO was added to dissolve the MTT-formazan crystal generated by live cells for 20 min in the dark. The optical density of DMSO solution at 492 nm was measured using a microplate reader (Thermo, Multiskan FC). The cell viability (%) was determined by comparing the absorbance at 492 nm with control wells containing cells cultured with 10 μL PBS solution. The experiments were performed in quartets and data are presented as average ± SD (n = 4).

The cytotoxicity of empty PS and cRGD-PS in 10 μL PBS solution was evaluated in a similar way. The cells were incubated with blank polymersomes at a final concentration of 1 or 2 mg/mL in an atmosphere containing 5% CO_2_ for 48 h at 37 °C.

**Flow cytometry assays**

A549 and MCF-7 cells were seeded in a 6-well plate at a density of 1 × 10^6^ cells/well in an atmosphere containing 5% CO_2_ at 37 °C for 24 h. cRGD-PS-DOX, PS-Dox or Lipo-Dox in 50 μL PBS (concentration: 10 μg Dox·HCl equiv./mL) was added. After 4 h incubation, the cells were washed with PBS for two times, detached by 0.25% (w/v) trypsin and 0.03% (w/v) EDTA, centrifuged at 1000 × g for 3 min, washed twice with PBS and suspended in 500 μL PBS. Fluorescence histograms were immediately recorded with a BD FACS Calibur flow cytometer (Becton Dickinson, USA, excitation 488 nm and emission 560 nm), analyzed using Cell Quest software based on 10,000 gated events. A549 cells cultured in 50 μL PBS solution were used as a control.

**Confocal microscopy measurements**

A549 cells were seeded on round glass coverslips in 24-well plates at a density of 1 × 10^5^ cells/well in an atmosphere containing 5% CO_2_ at 37 °C for 24 h. 50 μL cRGD-PS-Dox, PS-Dox or Lipo-Dox in phosphate buffer saline (PBS) was added (dosage: 10 μg Dox·HCl /mL). After 4 h, the culture medium was removed, replaced by fresh medium and the cells were further incubated for another 4 h. The culture medium was removed and the cells on microscope plates were washed three times with PBS, fixed with 4% paraformaldehyde solution for 15 min, and stained with fluorescein isothiocyanate labeled phalloidin (phalloidin-FITC) for 1 h. The cell nuclei were stained with 4′,6-diamidino-2-phenylindole (DAPI) for 10 min. The fluorescence images were obtained using a confocal microscope (TCS SP5, Leica Microsystems CMS GmbH, Germany).

**Table S1.** Tumor-to-Normal Tissue (T/N) distribution ratios of Dox·HCl at 12 h post-injection.

| Formulations | Heart | Liver | Spleen | Lung | Kidney |
| --- | --- | --- | --- | --- | --- |
| cRGD-PS-Dox | 6.34 ± 1.04 | 0.78 ± 0.13 | 1.56 ± 0.25 | 2.97 ± 0.48 | 2.29 ± 0.37 |
| PS-Dox | 2.17 ± 0.66 | 0.31 ± 0.09 | 0.66 ± 0.20 | 1.32 ± 0.40 | 0.82 ± 0.25 |
| Lipo-Dox | 2.58 ± 0.69 | 0.37 ± 0.10 | 0.80 ± 0.21 | 1.38 ± 0.37 | 1.08 ± 0.29 |

**Figure S1.** *In vitro* Dox release profiles of cRGD-PS-Dox in PB with 2 µM, 2 mM, 5 mM and 10 mM GSH at 37 °C. Data are presented as mean ± SD (n = 3).

**a**

**b**

**Figure S2. a** Viability of A549 cells after 4 h incubation with cRGD-PS-Dox plus 44 h culture in fresh media. Data are presented as the average ± SD (n = 4). **b** Flow cytometry of A549 cells after 4 h incubation with different formulations (10.0 μg/mL Dox·HCl).

**Figure S3.** Change of nude mice body weight in time following a single *i.v.* injection of cRGD-PS-Dox (DOX dose: 100 or 150 mg/kg) and Lipo-Dox (DOX dose: 10 or 20 mg/kg).

**REFERENCES**

1. Meng H, Zou Y, Zhong P, Meng F, Zhang J, Cheng R *et al.* A smart nano-prodrug platform with reactive drug loading, superb stability, and fast responsive drug release for targeted cancer therapy. *Macromol. Biosci.* **17**, 1600518 (2017).

2. Fang Y, Yang W, Cheng L, Meng F, Zhang J, Zhong Z. EGFR-targeted multifunctional polymersomal doxorubicin induces selective and potent suppression of orthotopic human liver cancer in vivo. *Acta Biomater.* **64**, 323-333 (2017).

3. Zou Y, Meng F, Deng C, Zhong Z. Robust, tumor-homing and redox-sensitive polymersomal doxorubicin: A superior alternative to Doxil and Caelyx? *J. Control. Release* **239**, 149-158 (2016).
